# Editorial: Novel circulating biomarkers and radiomics in pancreatic cancer

**DOI:** 10.3389/fonc.2023.1259070

**Published:** 2023-08-08

**Authors:** Shuai Ren, Rong Chen, Zhongqiu Wang

**Affiliations:** ^1^ Department of Radiology, Affiliated Hospital of Nanjing University of Chinese Medicine, Nanjing, China; ^2^ Department of Diagnostic Radiology and Nuclear Medicine, School of Medicine, University of Maryland, Baltimore, MD, United States

**Keywords:** pancreatic cancer, radiomics, circulating biomarkers, miRNA - microRNA, computed tomography (CT)

Pancreatic cancer (PC) is a highly lethal malignancy with a 5-year survival rate of < 9%. Approximately 80% of patients with advanced or metastatic PC suffer from an extremely poor prognosis, and the diagnosis of PC is generally at an advanced stage, which also presents a major challenge for clinical treatment, especially when the tumor has metastasized to other organs and proliferated to the extent that adequate surgical resection cannot be performed. Therefore, accurate PC diagnosis and treatment strategies are urgently needed in the early developmental stage. Due to its limited specificity, carbohydrate antigen 19-9 (CA19-9) is generally not recommended for early screening of PC. As the discovery of new serum biomarkers has recently received more attention, its combined detection of PC with CA19-9 has also been explored. Liquid biopsies, including circulating tumor cells (CTCs), circulating tumor DNA (ctDNA), microRNAs, and exosomes in blood, as well as biomarkers in urine and saliva, have been increasingly used in PC diagnosis. In addition, new technologies including radiomics, and the generation of mineable high-throughput data from medical images, have also provided a deeper understanding of pancreatic tissue heterogeneity, which holds great promise for the early diagnosis of PC.

This edition of *Frontiers in Oncology* aims to highlight the impact of novel circulating biomarkers and radiomics in the early detection of PC. Advances in new therapeutic strategies for PC have increased the opportunities for tumor downstaging. After peer review, a total of 4 wonderful works were published in this Research Topic, all of which were original studies. The studies were conducted in Germany, the Netherlands, Italy, and China respectively with a total of 40 contributing authors.

Different from traditional practice where medical images are solely treated for visual interpretation, Radiomics appears to offer a nearly limitless imaging biomarkers which helps to make clinically effective and cost-effective contributions to cancer care and serve as a decision-making tool for personalized medicine. Van Der Kroft et al. found that it is feasible to implement a data-driven radiomics approach to body composition imaging, and they were capable of extracting radiomics features which held improved predictive value compared to conventional body composition variables for the prediction of overall survival of pancreatic ductal adenocarcinomas (PDAC) patients undergoing primary pancreatic resection. Their research holds great promise in identifying patients who are susceptible to respond negatively to surgical treatment based on body composition, which facilitates personalized treatment strategies making or non-surgical treatment alternatives pursuing. Xu X. et al. sought to investigate the prognostic value of MRI-based radiomics nomogram in PDAC. As a result, they found that the radiomics nomogram incorporating the radiomics data, clinical data and TNM information exhibited precise survival prediction for PDAC, which may help accelerate personalized precision treatment. Their research sheds new light on accurate prediction of prognosis of PDAC patients, which facilitates treatment management planning; And it could also help to avoid aggressive treatments if poor survival is detected at the diagnosis. Xu J.-X. et al. explored clinical characteristics, radiological features and biomarkers of pancreatic metastases of small cell lung carcinoma (PM-SCLC), and established a convenient nomogram diagnostic predictive model to differentiate PM-SCLC from PDAC preoperatively. Their research provides a new inspiration in accurate preoperative diagnosis of PM-SCLC and differential diagnosis with PDAC, which facilitates in clinical decision making and patient prognosis prediction.

MicroRNAs (miRNAs) can be dysregulated in different types of cancers and the roles of miRNAs turn out to function to either tumor promoters or tumor suppressors. Notably, numerous potential miRNAs for the diagnosis and prognosis have been put forward, providing a new perspective on cancer screening. Bocchini et al. aimed to identify circulating and measurable prognostic miRNAs associated with ^18^F-FDG PET/CT status, higher risk and lower response to peptide receptor radionuclide therapy (PRRT) in gastro-entero-pancreatic neuroendocrine tumors (GEP-NETs), assessing their accuracy as prognostic biomarkers to improve the clinical management of PanNET patients. They concluded that hsa-miR-5096 performs well as a biomarker for ^18^F-FDG PET/CT and as independent predictor of progression-free survival (PFS). Besides, exosome-mediated delivery of hsa-miR-5096 may promote somatostatin receptor 2 (SSTR2) heterogeneity and thus resistance to PRRT. Their research leads to a candidate prognostic and low-complexity miRNA signature easily retrievable in plasma of PanNET patients, which facilitates in PanNET stratification treated with PRRT.

This Research Topic finally published 4 wonderful works, which investigated the potential value of radiomics and serum circulating miRNA in cancer imaging. However, the field of radiomics is still in lack of standardized evaluation of both the scientific integrity and the clinical relevance of the numerous published radiomics investigations resulting from the rapid growth of this area. Another core issue with regard to radiomics field is the decipher of the radiomics features in specific context, and how to give readers a description of radiomics features with classical imaging terminology, such as global hypodensity, heterogeneity, or possible different MRI signals. Radiologists should strive to improve the optimization of algorithms, the performance of constructed models, and language simplicity in decipher of radiomics.

## Author contributions

SR: Writing – original draft. RC: Writing – review & editing. ZW: Writing – original draft, Writing – review & editing.

